# The Preferred Treatment of Sternoclavicular Joint Infections: A Systematic Review

**DOI:** 10.7759/cureus.9963

**Published:** 2020-08-23

**Authors:** Barkat Ali, Venus Barlas, Anil K Shetty, Christopher Demas, Jess D Schwartz

**Affiliations:** 1 Surgery, University of New Mexico Health Sciences Center, Albuquerque, USA; 2 Surgery, University of New Mexico School of Medicine, Albuquerque, USA

**Keywords:** sternoclavicular joint infection, surgical management, joint resection, muscle flap

## Abstract

The treatment of sternoclavicular joint infection is a topic of controversy. This systematic review aims to evaluate the preferred treatment of sternoclavicular joint infections. A literature search using PubMed/MEDLINE®/Embase databases was conducted to identify publications on the surgical management of sternoclavicular joint infections. Case reports and studies without surgical management were excluded. The outcomes of interest included patient demographics, comorbidities, infectious etiologies, radiographic features, surgical management, and complications. Sixteen articles met the inclusion criteria. The mean age of the subjects was 53.4 years; there was a predominance of males (65%), and a minority of the subjects were obese (15%). The most common infectious etiology was methicillin-susceptible *Staphylococcus aureus* (MSSA) (48%). CT scan was reported in 46% of cases. The most common treatment was surgical resection of the joints (85%), followed by muscle flap closure of the wounds (54.2%). The complication rate ranged from 0-40%. Specifically, recurrence of infection was low with resection of the joint, followed by muscle flap closure. Given the heterogeneity of the methodology and inconsistency in the outcomes, a meta-analysis could not be performed. Overall, the current literature favors the resection of the sternoclavicular joint as the gold standard treatment. Closure of the wound using muscle flap seems to adequately treat this problem without any major untoward events.

## Introduction and background

Sternoclavicular joint infection is a rare disease. The true incidence rate of the condition is unknown, and it has an estimated prevalence of less than 1% [1,2]. The management of sternoclavicular joint infection, for the most part, remains controversial. The management ranges from nonoperative methods with intravenous antibiotics to invasive surgical interventions. Also, there is a wide variation in the surgical treatment options (i.e., ranging from incision and drainage, debridement and curettage, to radical resection of the sternal clavicular joint) [3,4]. Furthermore, following resection of the sternoclavicular joint, there are many different reconstructive options, ranging from primary closure to different types of muscle flaps [1,5,6]. Given the rare nature of the disease, studies have been limited to individual case series.

The aim of this systematic review was to identify the quality of the available literature regarding surgical management of this disease and to examine the patient demographics, comorbidities, symptoms, radiographic features, surgical treatment options, and outcomes associated with it.

## Review

Methods

Literature Search

A literature search using PubMed/MEDLINE®/Embase databases was conducted to identify publications regarding surgical management of sternoclavicular joint infections. The literature search was performed using appropriate keywords in English: "surgery," "treatment," "surgical intervention," "sternoclavicular joint infection," and "sternoclavicular joint septic arthritis."

Study Selection

We performed this systematic literature review according to the Preferred Reporting Items for Systematic reviews and Meta-analyses (PRISMA) statement guidelines [7]. Studies were selected for inclusion based on the following criteria: (1) the study reported surgical management of sternoclavicular joint infection; and (2) the studies were original studies. Studies were excluded if they: (1) did not involve surgical management of sternoclavicular joint infections; (2) were single case reports; (3) were written in a foreign language; and (4) were not available as full-length texts [8,9].

Data Extraction

Abstracts found using the aforementioned search terms were screened for eligibility by two independent reviewers. If consensus could not be reached, a third reviewer was asked to adjudicate. Full transcripts of the selected studies were then critically assessed for patient characteristics and clinical endpoints. Data extracted included study year, sample size, study design, patient characteristics, demographics, comorbidities, clinical data, radiographic data, microbiology, surgical management, and the complications associated with surgical management.

Outcome Measures

Outcomes measures of interest were overall complications, recurrence of osteomyelitis, mortality, hospital length of stay, and follow-up duration.

Assessment of the Level of Evidence

We used the Jovell and Navarro-Rubio classification to characterize the quality, quantity, and consistency of the studies, as shown in Table 1 [10]. We used this taxonomy to determine the quality of the level of evidence in order to assess the strength of the recommendation.

**Table 1:** Classification of study design

Level	Strength of evidence	Type of study design
I	Good	Meta-analysis of randomized controlled trials
II		Large-sample randomized controlled trials (n = ≧25 for each group)
III	Good to fair	Small-sample randomized controlled trials (n = <25 for each group)
IV		Non-randomized controlled prospective trials
V		Non-randomized controlled retrospective trials
VI	Fair	Cohort studies
VII		Case-control studies
VIII	Poor	Non-controlled clinical series; descriptive studies
IX		Anecdotes or case reports

Assessment of Methodological Quality

Two independent reviewers assessed the methodological quality of each study using the Coleman methodology score system, as shown in Table 2 [11].

**Table 2:** Mean Coleman methodology score

Components of the Coleman score (maximum score)	Individual components (score)
Study size (10)	>60 (10)
	41-60 (7)
	20-40 (4)
	<20, not stated (0)
Mean duration of follow-up (5)	>24 (5)
	12-24 (2)
	<12, not stated or unclear (0)
Number of different surgical procedures included in each reported outcome (10)	1 surgical procedure only (10)
	>1 surgical procedure, but >90% undergoing one procedure (7)
	Not stated, unclear, or <09% undergoing 1 procedure (0)
Type of study (15)	Randomized control study (15)
	Prospective cohort study (10)
	Retrospective study (0)
Diagnostic certainty (5)	In all (5)
	In >80% (3)
	In <80% (0)
Description of surgical procedure (5)	Adequate (5)
	Fair (3)
	Inadequate (0)
Description of postoperative rehabilitation (10)	Well described, >80% complying (10)
	Well described with 60%-80% complying (5)
	Protocol not reported or <60%-80% complying (0)

Assessment of Meta-Analysis

Given the small number of available studies with small individual sample sizes, we did not think performing a meta-analysis was feasible. This was further reinforced by the wide heterogeneity of surgical treatments, unclear description of surgical procedures, and inconsistency in reporting complications.

Results

Origins of Included Articles

**Figure 1:**
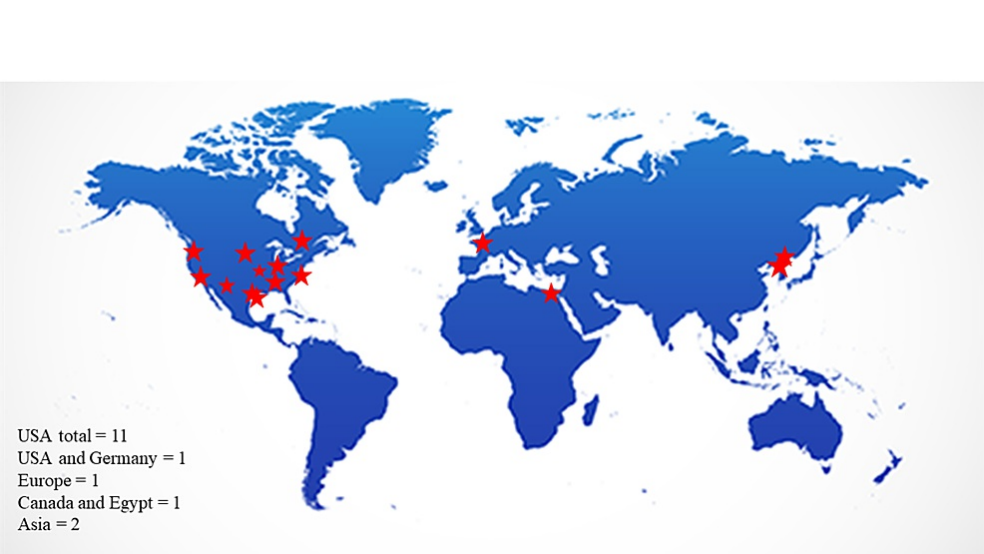
Origin of studies

As shown in Figure 1, most of the original articles on the surgical management of sternoclavicular joint infections came from the United States (11, 68%) [1,3,5,6,12-18]. Two studies came from Asia (12.5%) [4,19], one was from Europe (6.25%) [20], and two were multi-national [21,22].

Flowsheet Illustrating the Study Selection Process

**Figure 2:**
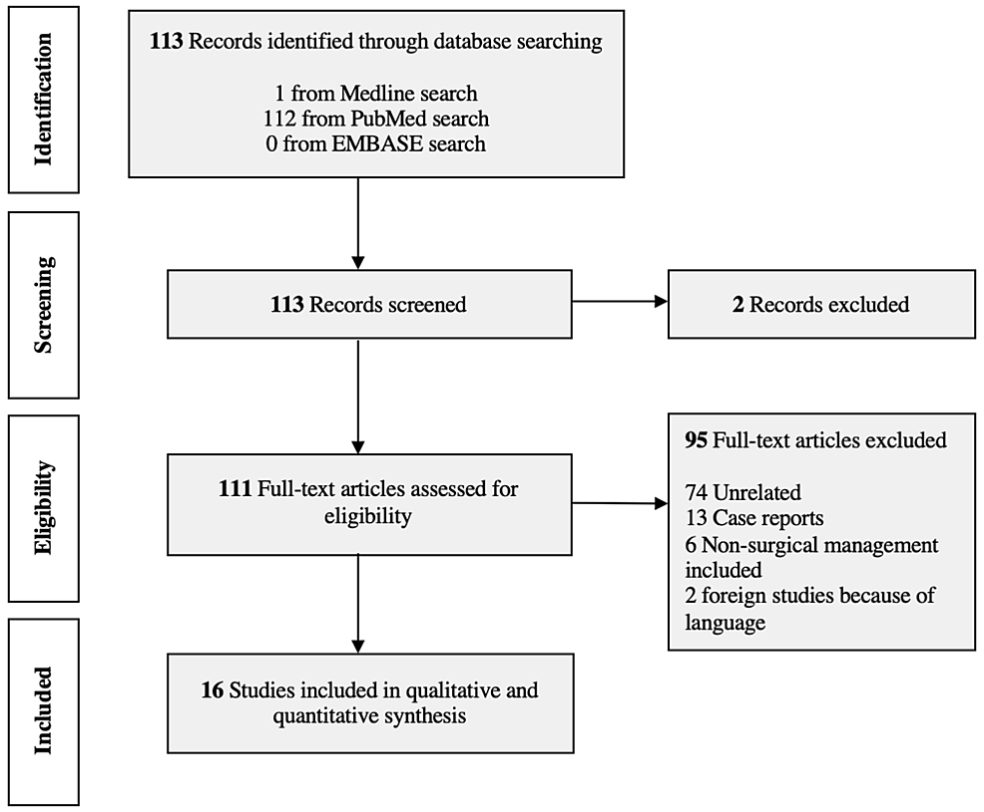
Flowsheet for study selection

Our electronic database search identified a total of 113 articles without duplicates. We removed two records as the publications were not peer-reviewed, and 111 titles and abstracts were screened. Of these articles, 95 full-text articles were excluded after assessing for eligibility, and 16 met the inclusion criteria for a systematic review, as shown in Figure 2.

Study Characteristics

We included a total of 16 studies pertaining to the surgical management of sternoclavicular joint infections (Tables 3, 4). The sum aggregate of sample size was 264 patients, with a range of 5-50. All of these studies were retrospective. Only one study, by Ali et al., was statistically powered given its largest sample size among all and would qualify for level VI evidence [1]. The rest were all descriptive studies and would qualify for level VIII evidence. We did not include individual case reports. The Coleman methodology scores ranged from 5-42. The diagnosis was certain in all studies. The description of surgical procedures was not clear enough in 13 of the 16 studies to distinguish between incision and drainage, debridement and curettage, and formal resection of the joint. The description of postoperative rehabilitation was also not clearly stated in any of the studies.

**Table 3:** Characteristics of studies

Author	Number of patients	Country	Journal	Timeframe	Coleman methodology score	Total
Ali et al. (2019) [1]	50	USA	Seminars in Thoracic and Cardiovascular Surgery	2004-2018	7, 5, 10, 0, 5, 5, 10	42
Jang et al. (2019) [4]	22	Korea	Infectious Diseases (London, England)	2009-2016	4, 5, 10, 0, 5, 5, 0	29
Von Glinski et al. (2019) [21]	13	USA/Germany	Journal of Clinical Orthopaedics and Trauma	2008-2015	0, 0, 10, 0, 5, 3, 0	18
Murga et al. (2017) [12]	15	USA	The Journal of Thoracic Disease	2001-2014	0, 0, 10, 0, 5, 3, 0	18
Kachala et al. (2016) [13]	40	USA	The Annals of Thoracic Surgery	1992-2012	4, 0, 10, 0, 5, 5, 10	34
Muesse et al. (2014) [14]	12	USA	Surgery Research and Practice	2002-2012	0, 0, 0, 0, 5, 3, 0	8
Chun et al. (2012) [19]	10	Korea	Journal of Shoulder and Elbow Surgery	1996-2008	0, 5, 10, 0, 5, 5, 10	35
Song et al. (2012) [6]	7	USA	The Annals of Thoracic Surgery		0, 5, 10, 0, 5, 5, 0	25
Abu Arab et al. (2011) [22]	14	Canada/Egypt	The European Journal of Cardio-Thoracic Surgery	2003-2009	0, 0, 0, 0, 5, 0, 0	5
Puri et al. (2011) [15]	20	USA	The Annals of Thoracic Surgery	2002-2009	4, 0, 10, 0, 5, 3, 0	22
Nusselt et al. (2011) [20]	5	Germany	Archives of Orthopaedic and Trauma Surgery	1992-2007	0, 0, 0, 0, 5, 0, 0	5
Bakaeen et al. (2008) [16]	5	USA	The American Journal of Surgery	1998-2006	0, 0, 0, 0, 5, 0, 0	5
Kendrick et al. (2007) [17]	7	USA	The American Surgeon	1997-2006	0, 0, 10, 0, 5, 3, 10	28
Ross et al. (2004) [3]	10	USA	Medicine (Baltimore)		0, 0, 0, 0, 5, 0, 0	5
Burkhart et al. (2003) [5]	26	USA	The Journal of Thoracic and Cardiovascular Surgery	1998-2001	4, 5, 7, 0, 5, 5, 10	36
Carlos et al. (1997) [18]	8	USA	The Journal of Thoracic and Cardiovascular Surgery	1994-1997	0, 5, 10, 0, 5, 5, 0	25

**Table 4:** Quality of studies and their conclusions SCJ: sternoclavicular joint; MCF: myocutaneous flap; DWVT: deep wound vacuum therapy; MRSA: methicillin-resistant *Staphylococcus aureus*

Study	Coleman methodology aggregate score	Conclusions
Ali et al. (2019) [1]	42	Wound closure with an MCF (primary or delayed) is associated with less recurrence of infections compared with DWVT closure. Radical resection of the entire SCJ with MCF (primary or delayed) should be considered the preferred management strategy in patients with SCJ infections
Burkhart et al. (2003) [5]	36	If no evidence of abscess or bone destruction is found, parenteral antibiotics should be initiated. When doubt persists as to the diagnosis, incision and drainage can be performed. However, when either abscess or bone destruction exists, advocate resection of the SCJ. Surgical resection combined with muscle transposition provides effective long-term outcome
Chun et al. (2012) [19]	35	Curative resection arthroplasty should be preferentially considered for patients with pyogenic infection of the SCJ
Kachala et al. (2016) [13]	34	Perform joint resection on all patients who can tolerate surgical intervention. Limited surgical intervention or joint aspiration may be warranted when the diagnosis is in question; however, once the joint infection is documented, believe resection is warranted to achieve optimal source control. Primary closure with a muscle flap can achieve similar outcomes to secondary intention in selected patients
Jang et al. (2019) [4]	29	Medical treatment alone or accompanied by limited surgery would appear to be successful therapeutic strategies for the complicated sternoclavicular septic arthritis caused by Staphylococcus aureus in selected patients that do not suffer from major complications. Surgery should be considered in patients with chest wall and/or neck abscesses
Kendrick et al. (2007) [17]	28	Initial treatment usually consists of antibiotics appropriate to the source of infection and results of blood cultures or needle aspirations, and control of the primary site of infection. Septic arthritis of the SCJ may be successfully treated in this fashion; however, the development of osteomyelitis necessitates surgical intervention. Failure of antibiotics to control fever and cellulitis leading to progression of the SCJ phlegmon or radiographic findings of osteomyelitis are evidence of refractory SCJ infection best managed surgically
Song et al. (2012) [6]	25	Aggressive surgical management including resection of the SCJ and involved ribs with pectoralis flap closure would appear to be the preferred treatment for all but the most minor infections of the SCJ. This approach has minimal impact on upper extremity function
Carlos et al. (1997) [18]	25	Most cases of early SCJ infections will respond to conservative measures. However, when radiographic evidence of infection beyond the SCJ is present, en bloc resection, although seemingly aggressive, results in immediate eradication of all infection with negligible functional morbidity. Prolonged antibiotic therapy or continued local drainage procedures appear to have little value in these cases, adding only to patient care costs and the potential sequelae of chronic infections
Puri et al. (2011) [15]	22	For SCJ infection, a single-stage resection and muscle advancement flap lead to a higher incidence of complications. Debridement with open wound care provides satisfactory outcomes with minimal perioperative complications but requires prolonged wound care
Von Glinski et al. (2019) [21]	18	Conservative management alone may suffice in early disease stages, knowing that a significant number of these patients will progress and require surgical intervention. However, recommend a thorough debridement plus SCJ resection followed by antibiotics (as soon as possible adapted to bacterial sensitivity)
Murga et al. (2017) [12]	18	Debridement of the joint is key to early diagnosis for cultures and early treatment. The sooner the patient can get to the operating room for debridement, the sooner the infection can be adequately treated to prevent a worsening infection. Early start of broad-spectrum antibiotics that provide coverage against MRSA infections and early surgical intervention is the ideal treatment. Surgical management should include incision, drainage, and joint resection. The joint should be resected in all cases
Muesse et al. (2014) [14]	8	Treat all patients who need surgical debridement for osteomyelitis of the SCJ with initial incision and debridement followed by two to three weeks of wound care along with antibiotic treatment followed by delayed resection of infected bone and pectoralis major muscle flap advancement into the acquired defect
Abu Arab et al. (2011) [22]	5	Surgery is indicated in cases of SCJ infections after the failure of an antibiotic therapy trial. The type of operation depends on the general condition of the patient and the presence or absence of osteomyelitis. SCJ resection is indicated when there is a recurrence of infection, sinus formation, severe osteomyelitis, and when there is no response to the other forms of surgical treatment
Nusselt et al. (2011) [20]	5	For sufficient infection control, surgical debridement of infected tissue combined with suitable antibiotic therapy is essential. The early stages of infection can be managed by simple incision, debridement, and drainage. In advanced stages of infection, a more radical intervention is preferable
Bakaen et al. (2008) [16]	5	SCJ infections in cirrhotic patients tend to be advanced and extensive in nature and pose a high surgical risk. Clinicians should have a high index of suspicion when a cirrhotic patient presents with SCJ pain in an effort to achieve an earlier diagnosis. Surgical drainage with adequate debridement may be better tolerated than a radical en bloc resection
Ross et al. (2004) [3]	5	If extensive bony destruction, chest wall phlegmon or abscess, retrosternal abscess, mediastinitis, or pleural extension is seen on imaging, en bloc joint resection, with debridement of bone and soft tissues until they appear healthy, is indicated. Small wounds can be allowed to heal by secondary intention. Larger wounds may require the involvement of the plastic surgeon to advance an ipsilateral pectoralis major muscle flap

Patient Characteristics

We calculated cumulative means and percentages based on the available data, as shown in Table 5. The total number of patients reported in these studies was 264. The mean age of the patients was 53.4 years; they were predominantly male (65%), and a minority was obese (BMI >30, 15%). The most common symptoms were pain, swelling, and fever (57%, 46%, and 19%, respectively). The most common comorbidities were diabetes mellitus, intravenous drug use, hypertension, and renal insufficiency (33%, 15%, 11%, and 6%, respectively). The most common diagnostic imaging was a CT scan with 46% reported. The most common organism on final wound cultures was methicillin-susceptible *staphylococcus aureus* (MSSA) (48%).

**Table 5:** Patient characteristics The outcome measure was not reported in the corresponding article for any cell without data DM: diabetes mellitus; IVDU: intravenous drug user; HTN: hypertension; CRF: chronic renal failure; CT: computed tomography; MSSA: methicillin-sensitive *Staphylococcus aureus*; MRSA: methicillin-resistant *Staphylococcus aureus*

Study	Sample	Mean age	Male	Obesity	Comorbidities				Symptoms			Imaging	Microbiology		
					DM	IVDU	HTN	CRF	Pain	Swelling	Fever	CT	MSSA	MRSA	Strep
Ali et al. (2019) [1]	50	49	37	14	25	17	20	2	50	50	22	50	25	5	6
Jang et al. (2019) [4]	22	61	17	3	5	2	-	-	-	-	-	-	-	-	-
Von Glinski et al. (2019) [21]	13	38	8	-	8	2	-	2	-	-	-	-	12	1	-
Murga et al. (2017) [12]	15	55	12	-	8	-	9	1	13	-	2	15	11	-	1
Kachala et al. (2016) [13]	40	57	28	14	11	4	-	4	37	30	18	37	23	6	4
Muesse et al. (2014) [14]	12	58	8	7	6	-	8	2	-	-	-	-	8	-	1
Chun et al. (2012) [19]	10	53	6	-	1	-	-	-	6	3	2	-	1	-	2
Song et al. (2012) [6]	7	53	5	-	2	-	-	-	7	7	-	-	1	-	1
Abu Arab et al. (2011) [22]	14	49	12	-	6	3	-	5	-	-	-	-	11	-	-
Puri et al. (2011) [15]	20	57	-	2	6	-	-	-	17	19	7	17	-	6	-
Nusselt et al. (2011) [20]	5	60	5	-	-	-	-	-	-	-	-	-	1	2	1
Bakaeen et al. (2008) [16]	5	57	5	-	2	-	-	-	-	-	-	5	-	1	3
Kendrick et al. (2007) [17]	7	59	5	-	4	-	5	-	-	-	-	-	7	-	-
Ross et al. (2004) [3]	10	45	9	-	2	3	-	-	-	-	-	-	6	2	1
Burkhart et al. (2003) [5]	26	-	-	-	-	-	-	-	21	14	-	-	17	-	-
Carlos et al. (1997) [18]	8	51	7	-	2		-	2	-	-	-	-	4	-	-
Total = 16 studies	264	801/15 (53.4)	164/264 (65%)	40/264 (15%)	88/264 (33.3%)	31/264 (11.7%)	42/264 (15.9%)	18/264 (6.8%)	151/264 (57.2%)	123/264 (46.6%)	51/264 (19.3%)	124/264 (46.9%)	127/264 (48.1%)	23/264 (8.7%)	20/264 (7.5%)

Preferred Treatment

**Figure 3:**
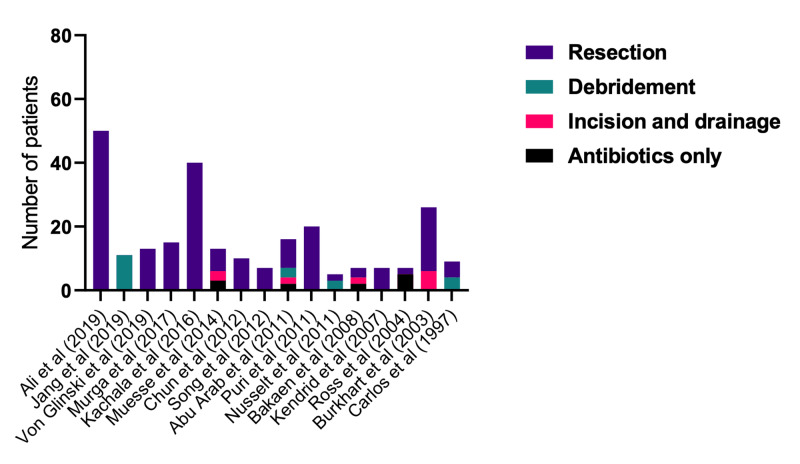
Treatment methods

There was considerable variation in surgical treatment in the available studies. However, resection of the entire sternoclavicular joint was the most commonly performed procedure (85% of the time) compared to debridement (9.7%) and incision and drainage (5.2%). Within the surgical management articles, there was a minority of patients who were treated with intravenous antibiotics alone (4.5%). We ranked studies according to their Coleman methodology scores, as shown in Figure 3. Apart from one study, most others appeared to recommend surgical resection of the entire sternoclavicular joint over antibiotic therapy alone [4].

Outcomes

Overall complications had a wide range of reporting, and five studies did not specify 30-day complications versus long-term complications. Additionally, two studies did not specifically clarify whether the mortality was related to sternoclavicular joint infection, related conditions, or remote unrelated causes. Overall, complication rates varied from 0% (Jang et al.) to 40% (Bakaeen et al. and Puri et al.) [4,15,16,21]. The mean follow-up ranged from three months (Von Glinski et al.) to 53 months (Jang et al.), as shown in Table 6 [4,21].

**Table 6:** Complications The outcome measure was not reported in the corresponding article for any cell without data

Study (n = sample size)	Overall complications	Muscle flap-related complications	Recurrence of infection	Deaths (30 days)	Follow-up (months)
Ali et al. (2019) [1]	11 (22%)	5 (10%)	6 (12%)	0	36
Jang et al. (2019) [4]	0	-	0	1 (5%)	53
Von Glinski et al. (2019) [21]	-	-	3 (23%)	2 (15%)	3
Murga et al. (2017) [12]	-	-	-	2 (13%)	-
Kachala et al. (2016) [13]	7 (17.5%)	-	4 (10%)	0	6.3
Muesse et al. (2014) [14]	-	-	-	-	-
Chun et al. (2012) [19]	1 (10%)	-	0	0	35.4
Song et al. (2012) [6]	0	-	-	-	28
Abu Arab et al. (2011) [22]	-	-	-	-	-
Puri et al. (2011) [15]	8 (40%)	-	-	1 (5%)	-
Nusselt et al. (2011) [20]	0	-	0	-	-
Bakaeen et al. (2008) [16]	2 (40%)	-	-	2 (40%)	-
Kendrick et al. (2007) [17]	-	-	-	0	5.2
Ross et al. (2004) [3]	-	-	1 (10%)	1 (10%)	-
Burkhart et al. (2003) [5]	2 (7.7%)	-	-	1 (3.8%)	25
Carlos et al. (1997) [18]	0	-	-	-	-

Recurrence of osteomyelitis was only reported in four studies with rates of 6% (Ali et al.), 10% (Kachala et al. and Ross et al.), and 23% (Von Glinksi et. al) [1,3,13,21]. Two of these studies further stratified the recurrence of infection by treatment modality and/or closure technique. Ali et al. reported a recurrence of infection in zero out of 25 patients treated with primary closure (0%), one out of 19 patients treated with delayed closure (5%), and two out of six patients treated with deep wound vacuum therapy alone (33%) [1]. Kachala et al. reported recurrence in one of 15 patients treated with primary closure (7%) and in three out of 25 patients treated with delayed closure (12%) [13].

Literature Quality and Risk of Bias

The majority of the reports did not provide high-quality evidence. The relatively small sample sizes and inconsistency in reporting of outcome measures rendered the studies not feasible for a meta-analysis.

Discussion

Infections of the sternoclavicular joint are rare, representing 0.5% of all joint infections seen, and the true incidence is unknown [1]. This is a separate clinical entity from sternomanubrial joint infections and/or post-sternotomy osteomyelitis following cardiac surgery procedures [2,3]. Many risk factors have previously been described, including intravenous drug use, diabetes mellitus, obesity, and renal failure [1,3-6]. In our review, we found that the clinical presentation varies greatly, often leading to a delay in diagnosis and resulting in a progression of the infection into deeper structures. If not identified early, patients will inevitably require surgical intervention in order to effectively treat and eradicate the infection. In this systematic review, we aimed to identify the demographics, initial presentation, and diagnostic workup of this condition, as well as compare different treatment modalities and the complications associated with them. After screening all articles related to sternoclavicular joint infections, we extensively reviewed 16 peer-reviewed full-text original studies that met the inclusion criteria. 

Initial patient presentations varied, considering the etiology of the infection, and morbidities. In general, however, the majority of patients presented with pain, swelling, or fever, though this was not a universal finding. Although a clinical diagnosis never seems to be an issue, results from bacterial cultures vary, and oftentimes there is no organism found in the final culture results. The most common organism that cultures did yield was MSSA, followed by methicillin-resistant *Staphylococcus aureus *(MRSA), *Streptococcus* species, and *Pseudomonas aeruginosa*. Consistent with the standard of care for osteomyelitis in other sites of the body, all the studies in this review reported the initial treatment plan including a total of four to six weeks of intravenous antibiotics regardless of the isolation of an organism on the final wound cultures [1,5,6].

Based on this review, it appears that the gold standard for the treatment of sternoclavicular joint infection should be surgical resection of the entire joint. This has been most widely adopted with the most favorable outcomes. Resection of the joint in addition to intravenous antibiotics eliminates the disease process effectively and expeditiously, as favored by the stronger studies in on this topic [1,3,12-16,18,19,22]. There was only one study where minimal debridement was compared with intravenous antibiotics alone and no resection of the joint at all. However, the study concluded that resection should be performed if there are neck and chest wall abscess [4].

The closure of the wound after resection of the sternoclavicular joint infection seems to favor using myocutaneous flaps, as evidenced by the stronger studies [1,5,6,22]. The use of myocutaneous flap can help obliterate the dead space resulting from resection of the sternoclavicular joint. By virtue of vascularized tissue, it can help clear the infection, which presumably translates into the promotion of healing and recovery. There was only one powered study that compared myocutaneous flap coverage to that of wound vacuum therapy. This paper demonstrated that myocutaneous flap closure was statistically superior to vacuum therapy alone [1].

Limitations to this review include our inability to include articles in foreign languages. Additionally, we point out our difficulty in performing a standard meta-analysis. Meta-analysis requires a quantitative integration of evidence from a number of related studies. For a rare disease such as sternoclavicular joint infections, the literature is limited to small series, and the data is often inconclusive. Hence, pooling the data adds strength to the study to help identify the best surgical management and complications associated with it. Unfortunately, the heterogeneity of the methodologies and inconsistency of outcome reporting we found in the literature precluded a formal standard meta-analysis.

This study does, however, provide a comprehensive review of the surgical management of this rare disease and provides a holistic picture of the current state of the literature, examines the quality of evidence, and leads towards a weighted conclusion

Additional citation pertaining to tables 1 and 2 (see correction notice): [23].

## Conclusions

The current literature favors resection of the entire sternoclavicular joint as the gold standard treatment in sternocleidomastoid joint infections. Furthermore, the authors conclude from this review that closure of the wound using muscle flap has been associated with the most favorable of outcomes, with the least amount of recurrent osteomyelitis and a reasonable flap-related complication rate.
