# Correction: The Preferred Treatment of Sternoclavicular Joint Infections: A Systematic Review

**DOI:** 10.7759/cureus.c42

**Published:** 2021-06-04

**Authors:** Barkat Ali, Venus Barlas, Anil K Shetty, Christopher Demas, Jess D Schwartz

**Affiliations:** 1 Surgery, University of New Mexico Health Sciences Center, Albuquerque, USA; 2 Surgery, University of New Mexico School of Medicine, Albuquerque, USA

This article has been corrected at the request of the authors to include an additional citation.

The following article was erroneously omitted from the original publication. While the original sources for tables 1 and 2 were correctly cited (citations 10 and 11), the concept was originated by Huang et al as seen in the following article: Huang K, Hollevoet N, Giddins G: Thumb carpometacarpal joint total arthroplasty: a systematic review. J Hand Surg Eur. 2015, 40:338-50. https://dx.doi.org/10.1177/1753193414563243

As a result the authors wish to include this citation in the published article and it has been added as the final citation (citation 23). Copyright licensing has also been obtained from the original publisher.

